# The use of machine learning to predict pharmacological therapy in gestational diabetes: A scoping review

**DOI:** 10.1111/dme.70171

**Published:** 2025-11-18

**Authors:** Jasmine R. Kirkwood, Natasha Galloway, Robert S. Lindsay, Areti Manataki, Deborah J. Wake, Rebecca M. Reynolds

**Affiliations:** ^1^ Centre for Cardiovascular Science, Queen's Medical Research Institute The University of Edinburgh Edinburgh UK; ^2^ Edinburgh Centre for Endocrinology and Diabetes NHS Lothian Hospitals Trust Edinburgh UK; ^3^ School of Cardiovascular and Metabolic Health The University of Glasgow Glasgow UK; ^4^ School of Computer Science University of St Andrews St Andrews UK; ^5^ Usher Institute The University of Edinburgh Edinburgh UK

**Keywords:** gestational diabetes mellitus (GDM), insulin, machine learning, oral agents, pharmacological therapy, prediction algorithms

## Abstract

**Aims:**

Early identification of pharmacological therapy for gestational diabetes mellitus (GDM), a common pregnancy complication, through machine learning could allow for better therapeutic strategies and improved treatment efficiency. This scoping review aimed to comprehensively review the machine learning models used to predict the need for pharmacological therapy in GDM.

**Methods:**

Four electronic databases—Embase, Medline, IEEE Xplore and Web of Science—were searched for publications between 1 July 2007 and 31 August 2024. Studies predicting pharmacological therapy for GDM using machine learning were included. The Joanna Briggs Institute and PRISMA‐ScR checklist was followed, and the Prediction model Risk Of Bias ASsessment Tool (PROBAST) was used to assess quality.

**Results:**

Included were 17 studies presenting 44 models, 61.4% (27/44) predicted any pharmacological therapy use and 38.6% (17/44) predicted insulin use alone. All were binary classifiers, and logistic regression was typically used. The overall area under the receiver operating curve had a median of 0.75. Common clinical variables were found to be predictors, such as history of GDM, gestational week at GDM diagnosis, pregestational body mass index, maternal age, HbA1c, fasting and 1 h glucose from 75 g oral glucose tolerance test. Though 65.9% of models were validated, there was a lack of external validation. There was no evidence of clinical application of the models.

**Conclusion:**

Logistic regression with common clinical variables was often used to predict pharmacological therapy for GDM. Few models were externally validated or clinically applicable.

**What's new?**

- Machine learning has been applied to many aspects of pregnancy care and diabetes.
- Logistic regression is a popular algorithm to predict pharmacological therapy for gestational diabetes. Most current models lack external validation, and as far as can be ascertained by a review of the published literature, none have been implemented clinically.
- All models are binary classifiers predicting either insulin or pharmacological therapy (grouping insulin and oral agents). Alongside external validation and clinical implementation, there is a gap in developing a multi‐class classifier to identify the risk for an oral agent or insulin for a person with gestational diabetes.

## 1 INTRODUCTION

Gestational diabetes mellitus (GDM) is one of the most common pregnancy complications, affecting 13.4% of live births worldwide in 2019^1^ and is rising in prevalence.^2^ Women with GDM typically attend multi‐disciplinary specialised clinics fortnightly, where their self‐monitoring blood glucose values (SMBG) and diet and exercise modifications are reviewed.^3^, ^4^ If blood glucose targets are unmet, some women will need medication, such as oral agents and/or insulin injections.^4^

GDM increases the risk of adverse pregnancy and neonatal outcomes if glucose levels are not well‐controlled.^5^, ^6^, ^7^ There is a 10‐fold increased risk of women with GDM developing subsequent type 2 diabetes,^8^ and a two‐fold increased risk of developing premature cardiovascular disease.^9^, ^10^, ^11^ In addition, children of women who had GDM are at a higher risk of obesity during their childhood and adolescence.^12^ Early identification of women with GDM who need pharmacological therapy could allow for more efficient therapeutic strategies for each woman and better allocation of resources.

Machine learning is a subset of artificial intelligence that uses algorithms to ‘learn’ from the data to optimise the performance metric,^13^ allowing insight into data that may elude human analysis. Machine learning has been used within healthcare alongside electronic health records for many different applications, including predicting and identifying diseases and treatments.^14^ In combination with the clinician's expertise, machine learning could give an opportunity to personalise the care of women with GDM and allow for prompt effective therapeutic recommendations, particularly within the remit of predicting pharmacological therapy.

Previous systematic reviews have identified risk factors for the need for pharmacological therapy to achieve good glycaemic control, but have not looked at the modelling algorithms or techniques used in detail.^15^, ^16^ Here we investigated the algorithms and their performance rather than just identifying risk factors.

This scoping review aimed to comprehensively review the methods, variables and quality in machine learning used to predict the need for prescribing or the escalation of pharmacological therapy in GDM, both at the start of or throughout pregnancy.

## 2 METHODS

The scoping review was conducted using the Joanna Briggs Institute (JBI) checklist,^17^ and the Preferred Reporting Items for Systematic Reviews and Meta‐Analyses—Scoping Review (PRISMA‐ScR) checklist^18^ and quality was assessed using the Prediction model Risk Of Bias ASsessment Tool (PROBAST).^19^ The protocol for the scoping review was registered on the Open Science Framework, website, using the JBI protocol template (osf.io/24dhk).

### 2.1 Search strategy

Four electronic databases: Embase, Medline, IEEE Xplore and Web of Science, were searched for published literature (Data S1). Electronic databases were chosen, and search terms were developed and refined in consultation with the study team and research librarian.

### 2.2 Inclusion and exclusion criteria

Studies published between 1 July 2007 and 31 August 2024, were included. The start date was chosen to be after the reporting of the Metformin in Gestational Diabetes trial,^20^ after this, Metformin was more widely used for GDM. Inclusion and exclusion criteria are shown in Table 1 using the JBI^17^ population, concept and context (PCC) framework. We included papers that had a population of women with GDM defined by the author and predicted pharmacological therapy for GDM using machine learning. As logistic regression could be classified as either a statistical or machine learning model, studies that reported predominantly statistical metrics were excluded.

**TABLE 1:** Inclusion and exclusion criteria using the population‐concept‐context framework.

	Inclusion criteria	Exclusion criteria
Population	Pregnant women who have GDM^a^	Non‐pregnant participants Pregnant women without GDM^a^ Women with T1DM^b^ or T2DM^c^
Concept	Predicting pharmacological therapy for GDM^a^ using machine learning	Predicting pharmacological therapy for GDM^a^ without using machine learning, or not predicting pharmacological therapy
Context	All patient groups and settings	
Types of studies	Any	Conference papers Review papers Editorial Commentary Letters Essays Books and book chapters
Language	English	Non‐English

^a^ GDM: Gestational diabetes mellitus.

^b^ T1DM: Type 1 diabetes mellitus.

^c^ T2DM: Type 2 diabetes mellitus.

### 2.3 Screening and selection of studies

**FIGURE 1:**
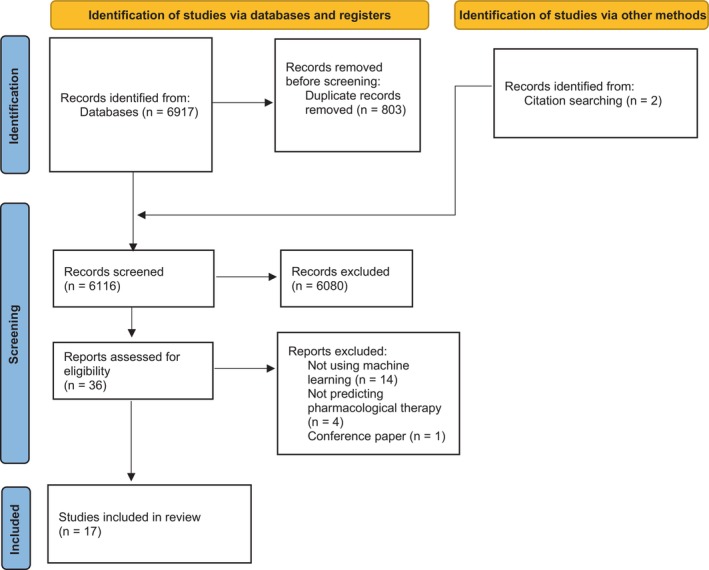
PRISMA‐ScR flowchart.

Using the search terms, 6917 studies were identified from the four databases and imported into Covidence.^21^ Two additional papers were added from the references of included papers, giving a total of 6919 studies identified. 803 were duplicates, leaving 6116 to be screened by their title and abstract. Screening and selection of the studies are shown in the PRISMA diagram in Figure 1. Title and abstract screening were completed by the first reviewer (JRK), and 9% (*n* = 531) were completed by the second reviewer (NG); 6080 studies were excluded, and 36 studies had a full‐text review. The full‐text review was completed by JRK and NG completed 11% (*n* = 4). Following the full‐text review, 19 studies were excluded, either for not using machine learning (*n* = 14), not predicting pharmacological therapy (*n* = 4) or being a conference paper (*n* = 1). This resulted in 17 studies being included in this scoping review. Disagreements between reviewers were resolved through discussion.

### 2.4 Charting the data

Data were extracted from the 17 included studies in Covidence^21^ and then exported and analysed using Microsoft Excel and R. The extracted data included general information on the study characteristics (title, author, country, research aims, study design, length of study and population), treatment predicted, GDM diagnosis criteria, variables, algorithms, performance and validation of the models. Studies' quality was assessed using the PROBAST checklist focusing on risk of bias and applicability within four domains: participants, predictors, outcomes and analysis.

For analysis, the models were grouped into two treatment prediction categories. These were (1) predicting pharmacological therapy, all of which were a change from diet, and (2) predicting insulin, which was a change from either diet or oral agents.

Due to a large proportion of the models (45.5%, 20/44), coming from Liao et al.^22^ a sensitivity analysis was performed excluding this study.

## 3 RESULTS

### 3.1 Overview

A total of 17 studies were included, as shown in the PRISMA flowchart (Figure 1), which were published between 2016 and 2023. An overview of the characteristics of the included studies is shown in Table 2 (a detailed summary of the included studies' characteristics is provided in Data S2). Published studies included participants located in Europe (25.3%, 6/17)^23^, ^24^, ^25^, ^26^, ^27^, ^28^; Asia (23.4%, 4/17)^29^, ^30^, ^31^, ^32^; North America (17.6%, 3/17)^22^, ^33^, ^34^; Australia (11.8%, 2/17)^35^, ^36^ and South America (5.9%, 1/17).^37^, ^38^ One study was a secondary analysis of a prospective observational study (5.9%, 1/17)^25^ and the rest were cohort studies, either retrospective (76.5%, 13/17)^23^, ^24^, ^27^, ^28^, ^29^, ^30^, ^31^, ^32^, ^33^, ^34^, ^36^, ^37^, ^38^; prospective (2/17, 11.8%)^26^, ^35^ and population‐based (5.9%, 1/17).^22^

**TABLE 2:** Summary of study characteristics.

	Author	Country	Type of prediction study	GDM^a^ diagnostic criteria	Data collection period (years)	Number of participants	Algorithm(s) described in the paper	Number of models described in the paper
Predicting Pharmacological therapy	Feghali et al.^33^	United States	Development and validation	Carpenter and Coustan's criteria	3	1174	Logistic regression	2
	Liao et al.^22^	United States	Development and validation	Carpenter and Coustan's criteria	10	30,474	CART^e^, LASSO^f^, Simple super learner^g^ Complex super learner^h^ Logistic regression	20
	Velardo et al.^23^	United Kingdom	Development and validation	IADSPG^c^ and national guidlines	5	1789	Logistic regression	1
	Yerlikaya et al.^24^	Austria	Development	IADSPG^c^	2	203	Logistic regression, Random Forest	4
Predicting insulin	Barnes et al.^35^	Australia	Development and validation	ADIPS^b^	23	3317	Logistic regression	1
	Ducarme et al.^25^	France	Development and validation	IADSPG^c^ and national guidelines	1	200	Logistic regression	1
	Eleftheriades et al.^26^	Greece	Development and validation	IADSPG^c^	8	775	CART^e^	1
	Ford et al.^36^	Australia	Development and validation	ADIPS^b^	1	2048	Logistic regression	1
	Harper et al.^34^	United States	Development and validation	Carpenter and Coustan criteria	6	360	Logistic regression	2
	Khin et al.^27^	United Kingdom	Development	IADSPG^c^	3	228	Logistic regression	1
	Nishikawa et al.^29^	Japan	Development	IADSPG^c^	1	529	Logistic regression	1
	Souza et al.^37^	Brazil	Development and validation	IADSPG^c^	3	408	Logistic regression	1
	Tamagawa et al.^30^	Japan	Development	IADSPG^c^ and national guidelines	9	388	Logistic regression	1
	Tang et al.^31^	China	Development	IADSPG^c^ and national guidelines	3	534	Logistic regression	1
	Watanabe et al.^32^	Japan	Development	IADSPG^c^	6	37	Logistic regression	1
	Weschenfelder et al.^28^	Germany	Development	IADSPG^c^ and national guidelines	5	454	Logistic regression	4
	Zaccara et al.^38^	Brazil	Development	ADA^d^	8	869	Logistic regression	1

^a^ GDM: Gestational diabetes mellitus.

^b^ ADIPS: Australasian Diabetes in Pregnancy Society.

^c^ IADPSG: International Association of the Diabetes and Pregnancy Study Group.

^d^ ADA: American Diabetes Association.

^e^ CART: Classification and regression Tree.

^f^ LASSO: Least absolute shrinkage and selection operator.

^g^ Simple super learner could have been included: response‐mean, least absolute shrinkage and selection operator regression, and classification and regression tree.

^h^ Complex super learner could have been response‐mean, least absolute shrinkage and selection operator regression, classification and regression tree, random forest and extreme gradient boosting.

**FIGURE 2:**
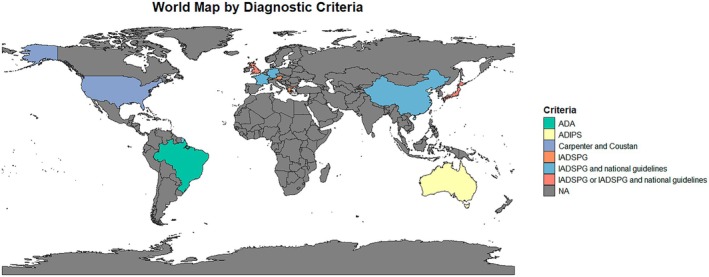
GDM diagnostic criteria and country of the included studies.

GDM was diagnosed with different diagnostic criteria, shown in Figure 2 alongside the country of the included studies. Mostly GDM was diagnosed with the International Association of the Diabetes and Pregnancy Study Groups (IADPSG) or IADPSG in combination with national guidelines, for example, the National Institute for Health and Care Excellence (NICE), (64.7%, 11/17),^23^, ^24^, ^25^, ^26^, ^27^, ^28^, ^29^, ^30^, ^31^, ^32^, ^37^ followed by the Carpenter and Coustan criteria (17.6%, 3/17),^22^, ^33^, ^34^ Australasian Diabetes in Pregnancy Society (ADIPS) (11.8%, 2/17),^35^, ^36^ American Diabetes Association (ADA) (5.9%, 1/17).^38^

From the 17 included studies, 44 models were described. Pharmacological therapy, either oral agents or insulin, was predicted by 61.4% (27/44) of the models,^22^, ^23^, ^24^, ^33^ of which 74.1% (20/27) were from Liao et al.^22^ The remaining 38.6% (17/44) predicted insulin, as a change from diet,^25^, ^26^, ^28^, ^29^, ^30^, ^31^, ^32^, ^35^, ^36^, ^37^, ^38^ glyburide^34^ or metformin.^27^

### 3.2 Study characteristics and population

The median duration for data collection was 5 years (range 1–23 years). There was a median per model of 1919 participants (range 37–30,474), with a median of 61.2% (range 30.2%–89.2%) of study participants per model in the control groups and 38.8% (range 10.8%–69.8%) in the prediction group. Only one study explicitly reported a sufficient sample size.^28^ However, using the commonly used calculation of one variable for ten adverse outcomes,^39^ it was found that all but one model^32^ had a sufficient sample size.

Ethnicity was reported in 41.2% (7/17)^22^, ^23^, ^27^, ^33^, ^34^, ^35^, ^36^ studies. Common exclusion criteria included multi‐fetal pregnancies (58.8%, 10/17)^25^, ^26^, ^27^, ^28^, ^30^, ^35^, ^36^, ^37^, ^38^; having other types of diabetes, (58.8%, 10/17)^22^, ^23^, ^25^, ^26^, ^30^, ^31^, ^32^, ^36^, ^38^ and missing data (47.1%, 8/17).^23^, ^24^, ^25^, ^27^, ^28^, ^35^, ^37^, ^38^

### 3.3 Clinical implementation

None of the identified studies had implementation in a clinical setting. One study developed a nomogram for clinical interpretation,^37^ and another,^23^ indicated that it could potentially be integrated into their existing mHealth app.

### 3.4 Algorithm

Overall, the most used algorithm was logistic regression (59.1%, 26/44),^22^, ^23^, ^24^, ^25^, ^27^, ^28^, ^29^, ^30^, ^31^, ^32^, ^33^, ^34^, ^35^, ^36^, ^37^, ^38^ and this was also true for both prediction categories. Following was the classification and regression tree (CART) (11.4%, 5/44),^22^, ^26^ least absolute shrinkage and selection operator (LASSO) (9.1%, 4/44),^22^ simple super learner (either response‐mean, LASSO or CART) (9.1%, 4/44),^22^ complex super learner (either response‐mean, LASSO, CART, random forest or extreme gradient boosting) (9.1%, 4/44)^22^ and random forest (9.1%, 4/44).^24^

### 3.5 Variables

The most frequently used variables in the models overall were history of GDM (47.7%, 21/44), gestational week at GDM diagnosis (45.5%, 20/44), pregestational BMI (40.9%, 18/44) and maternal age (38.6%, 17/44). This was the same for predicting pharmacological therapy: history of GDM (51.9%, 14/27), gestational week at GDM diagnosis (51.9%, 14/27), pregestational BMI (48.1%, 13/27) and maternal age (41.8%, 13/27). However, for predicting insulin, this differed, with 1 h glucose in the 75 g OGTT (64.7%, 11/17), fasting glucose in the 75 g OGTT (58.8%, 10/17), history of GDM (41.2%, 7/17) and HbA1c at GDM diagnosis (41.2%, 7/17) being the most frequently used model variables.

### 3.6 Performance metrics

**FIGURE 3:**
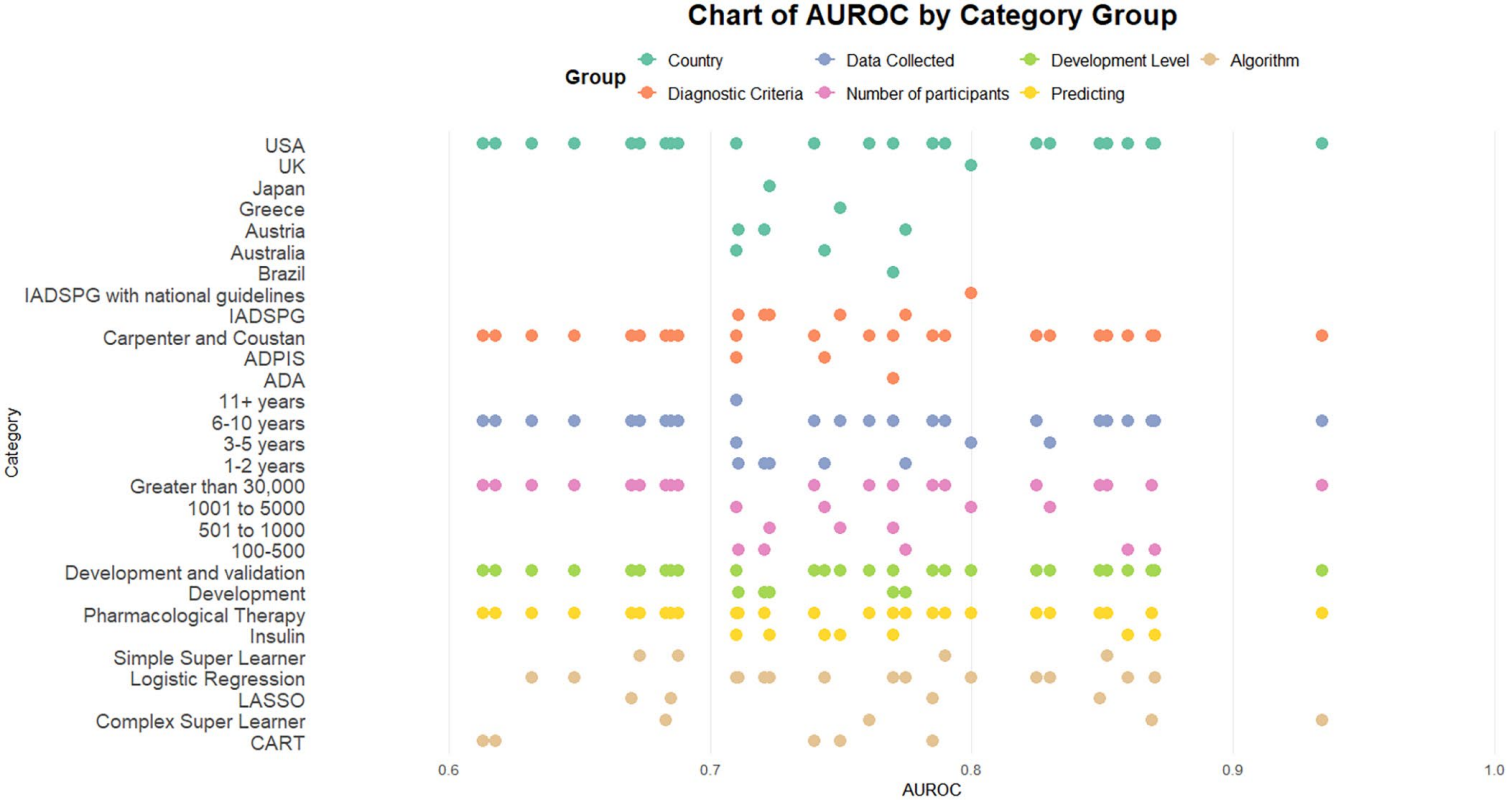
AUROC whole model performance for categories grouped by country, GDM diagnosis criteria, length of data collection and number of participants within the study, development level of the model, the prediction of the model and the algorithm used. ADA, American Diabetes Association; ADIPS, Australasian Diabetes in Pregnancy Society; AUROC, area under the receiver operating characteristics; CART, classification and regression tree; IADSPG, International Association of the Diabetes and Pregnancy Study Group; LASSO, least absolute shrinkage and selection operator; UK, United Kingdon; USA, United States of America. Simple super learner could have been included response‐mean, least absolute shrinkage and selection operator regression, and classification and regression tree, Complex super learner could have been response‐mean, least absolute shrinkage and selection operator regression, classification and regression tree, random forest and extreme gradient boosting.

The models were evaluated through several different methods, including the area under the receiver operating curve (AUROC) (95.5%, 42/44),^22^, ^23^, ^24^, ^25^, ^26^, ^28^, ^29^, ^30^, ^31^, ^32^, ^33^, ^34^, ^35^, ^36^, ^38^ sensitivity and specificity (36.4%, 16/44)^25^, ^27^, ^28^, ^29^, ^30^, ^31^, ^32^, ^33^, ^34^, ^35^, ^37^ and positive predictive values (PPV) and negative predictive value (NPV) (25.0%, 11/44).^25^, ^27^, ^28^, ^30^, ^33^, ^35^, ^37^ AUROC had a median of 0.75 (range 0.61–0.93) which did not exhibit considerable changes between algorithms, prediction pathways or GDM diagnostic criteria (Figure 3). It also did not seem affected by class imbalances (Data S3).

### 3.7 Model validation

Validation of the models was conducted on 65.9% (29/44) of the models. Validation methods included temporal,^22^, ^35^ geographical,^26^, ^35^ bootstrapping^34^ and cross‐validation, either 10‐fold,^22^, ^26^, ^33^ 5‐fold^23^, ^36^ or leave‐one‐out.^37^ The validation performance AUROC had a median of 0.72 (range 0.59–0.82); all models performed slightly worse on the validation set, but by no more than 0.12.

### 3.8 Quality assessment

**FIGURE 4:**
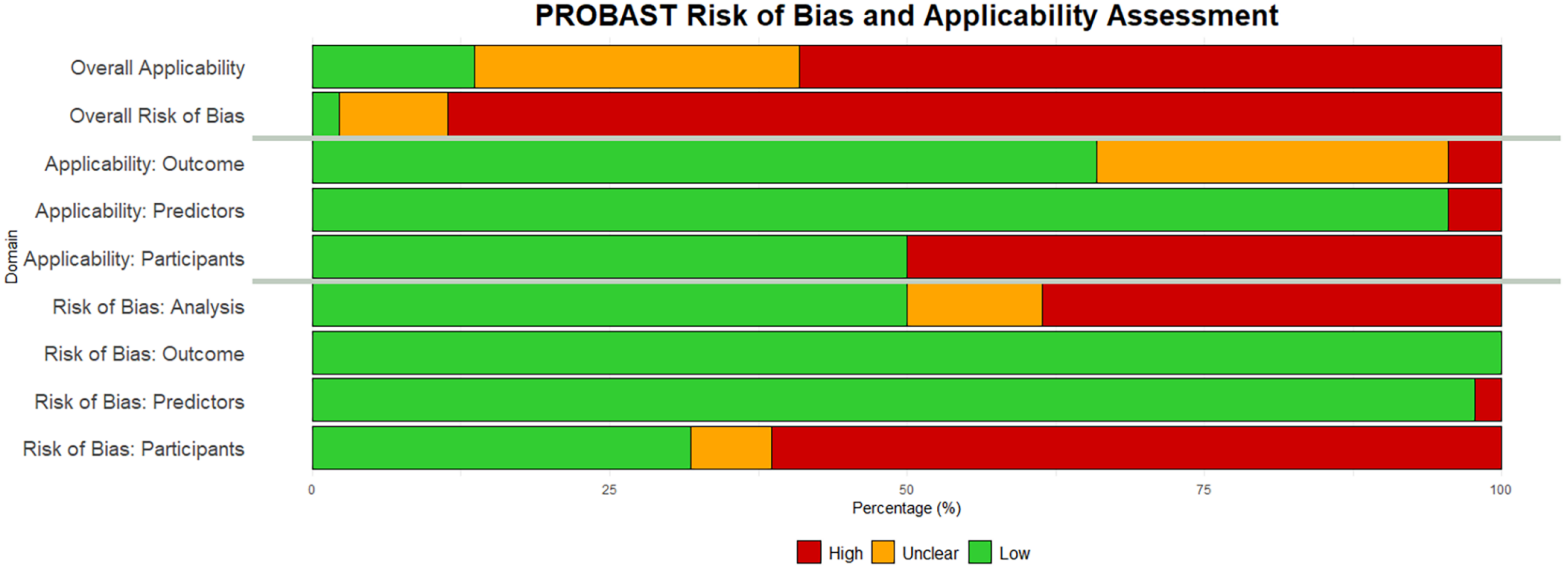
Risk of bias summary using PROBAST. The review authors' judgements on each risk of bias and applicability domains for all included studies in the analysis.

The PROBAST tool was used to assess the models' risk of bias and applicability within four domains: participants, predictors, outcomes and analysis; a summary of the results is shown in Figure 4 (a detailed summary is provided in Data S4). It was found overall that there was a predominantly high risk of bias (88.6%, 39/44; unclear: 9.1%, 4/44; low: 2.3% 1/44), and concern over applicability was varied (high: 59.1%, 26/44; unclear: 27.3% 12/44; low: 13.6% 6/44). The risk of bias between the models predicting insulin or those predicting pharmacological therapy was similar (high: 88.2% 15/17 vs. 88.9% 24/27, respectively). There was a higher concern over applicability in the pharmacological predicting group (77.8%, 21/27) compared with the insulin predicting group (29.4%, 5/17).

The high risk of bias introduced by the selection of participants, in 61.4% (27/44) of the models, was often because of the inclusion of women with pre‐diabetes or the use of SMBG data. There was a low risk of bias (97.7%, 43/44) introduced by the predictors, which were defined, assessed and available appropriately for the models, which led to a low concern (95.5%, 42/44) as well. All had a low risk of bias regarding the outcomes of the models; however, there were concerns about the outcomes (low: 65.9%, 292/44; unclear: 29.5%, 13/44), mostly due to the data used not matching the outcome described. Finally, half of the model analyses had a low risk of bias (high: 38.6%, 17/22; unclear: 11.4%, 5/44; low: 50.0%, 22/44); high and unclear risk of bias were due to unbalanced or small datasets, unclear approaches to missing data or inappropriate model assessment.

### 3.9 Sensitivity analysis

Liao et al.^22^ presented 20 models, using five different algorithms, (logistic regression, CART, LASSO, simple super learner, complex super learner) and four levels of data: (1) 1‐year preconception to last menstrual period; (2) last menstrual period to before diagnosis of GDM; (3) at the time of diagnosis of GDM and (4) 1 week after diagnosis of GDM (including SMBG data), involving 30,474 participants and 25 variables. As this skewed the results, a sensitivity analysis was conducted where Liao et al.^22^ was removed from the results (Data S5).

After the removal of Liao et al.,^22^ the percentage of models predicting insulin increased, (70.8%, 17/24) and the prediction of pharmacological therapy decreased (29.2%, 7/24). The median study period of 5 years (range 1–23) did not change. There was a reduction in the number of participants per model from 1919 participants to 304 (range 37–2217), an increase in the percentage of participants in the control group from 61.2% to 65.1% (range 30.2–89.2) and a slight decrease in the prediction group from 38.8% to 35.0% (range 10.8%–69.8%).

The study by Liao et al.^22^ was the only one to use LASSO, simple super learner and complex super learner; as a result, logistic regression became the most widely used (97.7%, 22/24), with CART and random forest used just once each (4.2%, 1/24).

Common predictive variables in the models were now 1 h glucose in the 75 g OGTT (58.3% 14/24), fasting glucose in the 75 g OGTT (54.2%, 13/24), maternal age (41.7%, 10/24) and gestational week at GDM diagnosis (41.7%, 10/24). There were also changes regarding the predictive variables for pharmacological therapy, which are now, maternal age (85.7%, 6/7), gestational week at GDM diagnosis (57.1%, 4/7), pregestational BMI (57.1%, 4/7) and parity (57.1%, 4/7).

AUROC was used to assess the performance of 91.7% (22/24) of the models, which had a minor decrease in the median from 0.75 to 0.74 (range 0.70–0.87). There was a large reduction in validated studies from 65.9% to 37.5% (9/24).

The overall risk of bias followed a similar pattern, (high: 79.2%, 19/24; unclear: 16.7%, 4/24; low: 4.2%, 1/24). However, the concerns over applicability became more unclear, (high: 25.0%, 6/24; unclear: 50.0%, 12/24; low: 25.0%, 6/24).

Overall, through the sensitivity analysis, it can be seen that Liao et al.^22^ were most influential in increasing the participant numbers, adding additional algorithms and altering predictive variables.

## 4 DISCUSSION

This review has comprehensively investigated the use of machine learning algorithms, variables and performance in predicting pharmacological therapy in GDM and assessed their quality. There was a total of 17 studies that described 44 models included. All the models were binary classifiers; none were multi‐class. We grouped them into two groups, predicting pharmacological therapy (61.4%, 27/44) and predicting insulin (39.6%, 17/44). The studies were published between 2016 and 2023, from a range of countries with different demographics and diagnosis guidelines; hence, the pooled result of this review is generalisable.

Overall, common clinical variables were used in the models, such as history of GDM, gestational week at GDM diagnosis, pregestational BMI and maternal age. After sensitivity analysis, these then included fasting and 1 h glucose values from the 75 g OGTT. This has some overlap with other reviews,^15^, ^16^ which identified variables and risk factors for the need for pharmacological treatment among women with GDM.

Logistic regression was the most popular applied algorithm. Logistic regression is easy to implement, interpretable, can be used on small data sets and is not computationally expensive; hence, it is often used in this medical setting.

The performance of the models was mostly evaluated by AUROC, which had a median of 0.75. AUROC was the only performance metric that was used across the different algorithms: logistic regression, CART, LASSO, simple super learner and complex super learner. Differences in data population and the use of logistic regression outweigh the other algorithms, making it challenging to determine the best‐performing algorithm. Nevertheless, logistic regression and CART performed well and could be a good starting point for future model development. In addition, it should be considered, that for this particular clinical scenario, it may be better to have an algorithm that gives more false positives than false negatives, as these would be overseen by a clinician.

It is important to acknowledge the geographic and ethnic differences in the pathophysiology and clinical presentation of GDM, which may lead to variation in modelling performance, hence the need for model validation across different GDM populations. A reasonable number of the models were validated; however, few were externally validated. The validation performance was good, indicating that the models are robust and generalisable. There does, however, need to be more external validation, particularly on data using different demographics to ensure that the models perform sustainably.

Using PROBAST^19^ to assess the quality of the models, overall, there was a high risk of bias and concerns over applicability. The issues were mainly due to women with pre‐diabetes being included in the modelling, unclear descriptions of approaches to missing data and the use of unbalanced and small datasets. These are areas that could be improved in future studies.

Machine learning has been shown to provide statistically significant improvements in health outcomes when incorporated into digital health interventions within real‐life studies.^40^ In a review by Sahota et al.^41^ of 36 randomised control trials of clinical decision support systems, 63% (22/35) of the studies showed an improvement in care; they found, however, that the improvements were in processes of care rather than patient outcomes. Equally importantly, they found no significant reduction in major patient morbidity or mortality, demonstrating that when machine learning is incorporated into a care system, it is unlikely to cause harm to patients. GDM care could be personalised through the incorporation of a pharmacological therapy prediction model, which could also help streamline care. For example, it could reduce the number of follow‐up appointments for women who have been identified as low risk for pharmacological therapy and allow for earlier more targeted intervention for high‐risk women. As predictions are not perfect, a model should be implemented alongside a monitoring system, to ensure the safety of all patients. Future work needs to be done on the implementation of such predictive models in care.

### 4.1 Limitations

The heterogeneity of the included studies, including the different GDM diagnostic criteria, inclusion and exclusion criteria and datasets available for each model limited the ability to make direct comparisons. These factors also contributed to simplified data extraction, and as such, the full complexities of the models may not be captured. Furthermore, due to the nature of a review, other relevant literature may have been published since the search.

## 5 CONCLUSION

The use of machine learning to predict pharmacological intervention in GDM could be easily implemented in clinics to risk‐stratify patients and therefore personalise care and allocate resources more appropriately. From this review, it was found that a popular approach was logistic regression that had a median AUROC of 0.75 and used clinically available variables such as history of GDM, gestational week at GDM diagnosis, pregestational BMI, maternal age, HbA1c, fasting and 1 h‐glucose in the 75 g OGTT. There were no multi‐class models presented. Furthermore, there was a lack of external validation, which future models would benefit from incorporating.

## FUNDING INFORMATION

JRK was funded by Medical Research Scotland (PHD‐50224‐2020). RMR acknowledges support from the British Heart Foundation (RE/18/5/34216).

## CONFLICT OF INTEREST STATEMENT

There are no conflicts of interest.

## Supporting information


Data S1.

Data S2.

Data S3.

Data S4.

Data S5.

## Data Availability

Data sharing not applicable to this article as no datasets were generated or analysed during the current study.
