# Supplementary material for: The use of machine learning to predict pharmacological therapy in gestational diabetes: A scoping review
Source: Diabet Med. 2025 Nov 18;43(2):e70171. doi: 10.1111/dme.70171 (PMC12857867; doi:10.1111/dme.70171)
Supplement: Supplementary file 1 — Data S1. [file DME-43-e70171-s002.docx]

Supplementary material 1

Search terms

# Embase

( (machine.mp. or *supervised/) adj2 learning.mp.

OR

((artificial or computer or machine) adj2 intelligence).mp.

OR

(Regression or classification or ai or ml).mp

OR

exp artificial intelligence/

OR

exp machine learning/)

AND

(((insulin or metformin or glyburide) adj2 (failure or pregan*or need)).mp.

OR

(prediction adj3 (metformin or insulin or glyburide or drug or pharmacological or model or diet)).mp

OR

exp metformin/ad, dt, pd [Drug Administration, Drug Therapy, Pharmacology]

OR

exp insulin/ad, pd, th [Drug Administration, Pharmacology, Therapy]

OR

exp glibenclamide/ad, pd [Drug Administration, Pharmacology]

OR

exp diet therapy/

OR

medication therapy management/

OR

exp glucose blood level/

OR

exp glucose level/)

AND

(((gestation* or pregan* or maternal) adj3 (diabete* or hyperglyc?emia or glucose)).mp.

OR

(Fasting blood glucose* or fgd or bg or gdm).mp

OR

exp pregnancy diabetes mellitus/dm, dt, th [Disease Management, Drug Therapy, Therapy]))

AND

limit XX to yr="2007-2024"

AND

limit XX to (english language and embase and (article-in-press status or embase status))

# IEEE Xplore

[manual] Filters:

2007 – 2024

Journals

((machine OR *supervised) NEAR/2 learning

OR

((artificial OR computer OR machine) NEAR/2 intelligence )

OR

Regression OR classification OR ai OR ml

OR

Artificial intelligence

OR

Exp machine learning )

AND

(prediction NEAR/3 (metformin OR insulin OR drug OR pharmacological OR model OR diet OR glyburide)

OR

((insulin OR metformin OR glyburide) NEAR/2 (failure OR pregan*OR need))

OR

exp Metformin/

OR

Insulin/

OR

Medication Therapy Management

OR

Exp Blood glucose drug therapy/ OR glycemic control)

AND

( (gestation* OR pregan* OR maternal) NEAR/3 (diabete* OR hyperglyc?emia OR glucose)

OR

Fasting blood glucose* OR fgd OR bg OR gdm

OR

diabetes, gestational/ OR pregnancy in diabetics/)

# Medline

((machine.mp. or *supervised/) adj2 learning.mp.

OR

((artificial or computer or machine) adj2 intelligence).mp.

OR

exp Artificial Intelligence/cl [Classification]

OR

exp Machine Learning/ or exp Algorithms/

OR

(Regression or classification or ai or ml).mp.

AND

( exp Drug Therapy/cl, mt [Classification, Methods]

OR

exp Glycemic Control/

OR

exp Metformin/ad, pd, tu [Administration & Dosage, Pharmacology, Therapeutic Use]

OR

exp Insulin/ad, pd, tu [Administration & Dosage, Pharmacology, Therapeutic Use]

OR

exp Diet Therapy/cl [Classification]

OR

exp Blood Glucose/ad, pd [Administration & Dosage, Pharmacology]

OR

exp Glyburide/ad, bl, pd [Administration & Dosage, Blood, Pharmacology]

OR

(prediction adj3 (metformin or insulin or glyburide or drug or pharmacological or model or diet)).mp

OR

((insulin or metformin or glyburide) adj2 (failure or pregan*or need)).mp

AND

( ((gestation* or pregan* or maternal) adj3 (diabete* or hyperglyc?emia or glucose)).mp.

OR

(Fasting blood glucose* or fgd or bg or gdm).mp

OR

Pregnancy in Diabetics/ or exp Diabetes, Gestational/

OR

exp Pregnancy in Diabetics/ or exp Diabetes, Gestational/ )

AND

((2007* or 2008* or 2009* or 2010* or 2011* or 2012* or 2013* or 2014* or 2015* or 2016* or 2017* or 2018* or 2019* or 2020* or 2021* or 2022* or 2023* or 2024*).ed. )

# Web of Science

(**(((TS=( (machine or supervised or unsupervised) NEAR/2 learning) OR TS=(((artificial or computer or machine) near/2 intelligence ))) OR TS=(Regression or classification or ai or ml ))))**

**AND**

**(TS=(prediction near/3 (metformin or insulin or glyburide or drug or pharmacological or model or diet)) or TS=(((insulin or glyburide or metformin) near (failure))) or**
**TS=(((insulin or metformin or glyburide) near (pregan*))) or**
**TS=(((insulin or metformin or glyburide) near (need))))**

**AND**

**(TS=( (gestation* or pregan* or maternal) NEAR/3 (diabete* or hyperglyc?emia or glucose)) or TS=(Fasting blood glucose* or fgd or bg or gdm ))**

MANUAL

Year ranges 2007-2024

Language English

Exclude all conference abstracts

Include articles, review articles
