# Supplementary material for: The use of machine learning to predict pharmacological therapy in gestational diabetes: A scoping review
Source: Diabet Med. 2025 Nov 18;43(2):e70171. doi: 10.1111/dme.70171 (PMC12857867; doi:10.1111/dme.70171)
Supplement: Supplementary file 2 — Data S2. [file DME-43-e70171-s001.docx]

Supplementary material 2

Summary table of study characteristics

Table - Summary of study characteristics

|  | Author | Country | Study design | Type of prediction study | GDM^a^ diagnostic criteria | Data collection period (years) | Number of participants | Algorithm used in each model described in the paper | The performance of the model | Aims relevant to review | Outcomes relevant to review |
| --- | --- | --- | --- | --- | --- | --- | --- | --- | --- | --- | --- |
| Predicting pharmacological therapy | Feghali et al. 2019 ^1^ | United States | Retrospective cohort study | Development and validation | Carpenter and Coustan criteria | 3 | 1174 | Logistic regression | AUROC^i^ 0.71 | To identify if the initial response to diet can predict the need for pharmacological therapy in women with GDM^a^ | Glucose values from the first week of diet were the strongest predictor of needing pharmacological therapy. |
|  |  |  |  |  |  |  |  | Logistic regression | AUROC^i^ 0.83 Sensitivity 72.2%,  Specificity 86.8%,  PPV^j^ 92.5%,  NPV^k^ 58% |  |  |
|  | Liao et al. 2022 ^2^ | United States | Population-based cohort study | Development and validation | Carpenter and Coustan criteria | 10 | 30474 | CART^e^ | AUROC^i^ 0.613 (0.603-0.622, 95% CI^l^) | To investigate whether clinical data at varied stages of pregnancy can predict GDM^a^ treatment modality. | Clinical data demonstrated reasonably high predictability for GDM^a^ treatment modality at the time of GDM^a^ diagnosis and high predictability at 1-week post GDM^a^ diagnosis. |
|  |  |  |  |  |  |  |  | CART^e^ | AUROC^i^ 0.618 (0.609-0.628 95% CI^l^) |  |  |
|  |  |  |  |  |  |  |  | CART^e^ | AUROC^i^ 0.740 (0.732-0.748, 95% CI^l^) |  |  |
|  |  |  |  |  |  |  |  | CART^e^ | AUROC^i^ 0.785 (0.777-0.792, 95% CI^l^) |  |  |
|  |  |  |  |  |  |  |  | LASSO^f^ | AUROC^i^ 0.670 (0.663-0.676, 95% CI^l^) |  |  |
|  |  |  |  |  |  |  |  | LASSO^f^ | AUROC^i^, 0.685 (0.678-0.691, 95% CI^l^) |  |  |
|  |  |  |  |  |  |  |  | LASSO^f^ | AUROC^i^ 0.785 (0.780-0.791, 95% CI^l^) |  |  |
|  |  |  |  |  |  |  |  | LASSO^f^ | AUROC^i^ 0.849 (0.845-0.854, 95% CI^l^) |  |  |
|  |  |  |  |  |  |  |  | Simple super learner^g^ | AUROC^i^ 0.673 (0.667-0.679, 95% CI^l^) |  |  |
|  |  |  |  |  |  |  |  | Simple super learner^g^ | AUROC^i^ 0.688 (0.682-0.695, 95% CI^l^) |  |  |
|  |  |  |  |  |  |  |  | Simple super learner^g^ | AUROC^i^ 0.790 (0.785-0.796, 95% CI^l^) |  |  |
|  |  |  |  |  |  |  |  | Simple super learner^g^ | AUROC^i^ 0.852 (0.848-0.857, 95% CI^l^) |  |  |
|  |  |  |  |  |  |  |  | Complex super learner^h^ | AUROC^i^ 0.683 (0.676-0.689, 95% CI^l^) |  |  |
|  |  |  |  |  |  |  |  | Complex super learner^h^ | AUROC^i^ 0.761 (0.756-0.767, 95% CI^l^) |  |  |
|  |  |  |  |  |  |  |  | Complex super learner^h^ | AUROC^i^ 0.869 (0.865-0.873, 95% CI^l^) |  |  |
|  |  |  |  |  |  |  |  | Complex super learner^h^ | AUROC^i^ 0.934 (0.931-0.936, 95% CI^l^) |  |  |
|  |  |  |  |  |  |  |  | Logistic regression | AUROC^i^ 0.632 (0.623-0.640, 95% CI^l^) |  |  |
|  |  |  |  |  |  |  |  | Logistic regression | AUROC^i^ 0.648 (0.640-0.656, 95% CI^l^) |  |  |
|  |  |  |  |  |  |  |  | Logistic regression | AUROC^i^ 0.770 (0.764-0.775, 95% CI^l^) |  |  |
|  |  |  |  |  |  |  |  | Logistic regression | AUROC^i^ 0.825 (0.820-0.830, 95% CI^l^) |  |  |
|  | Velardo et al. 2021 ^3^ | United Kingdom | Retrospective cohort study | Development and validation | IADSPG^c^ and national guidelines | 5 | 1789 | Logistic regression | Median AUROC^i^ 0.8 | To assess whether data collected through a mHealth system can be analysed to automatically evaluate the switch to pharmacological treatment from diet-based management of GDM. | Using real-time data collected via a mHealth system may further improve the timeliness of the intervention and potentially improve patient care |
|  | Yerlikaya et al. 2018 ^4^ | Austria | Retrospective cohort study | Development | IADSPG^c^ | 2 | 203 | Logistic regression | AUROC^i^ 71.1 | To assess the association between OGTT^m^ glucose levels and the requirement of pharmacotherapy in GDM^a^ patients classified by the IADPSG^c^ criteria. | OGTT^m^ glucose measures in addition to clinical risk factors showed promising properties for risk stratification in GDM^a^ patients classified by the recently established IADPSG^c^ criteria. |
|  |  |  |  |  |  |  |  | Logistic regression | AUROC^i^ 72.1 |  |  |
|  |  |  |  |  |  |  |  | Logistic regression | AUROC^i^ 77.5 |  |  |
|  |  |  |  |  |  |  |  | Random forest | Performance not reported. |  |  |
| Predicting insulin | Barnes et al. 2016 ^5^ | Australia | Prospective cohort study | Development and validation | ADIPS^b^ | 23 | 3317 | Logistic regression | AUROC^i^ 0.710 (0.675-0.745, 95% CI^l^) | To identify women with GDM who are more likely to require insulin therapy vs diet alone. | A validated model has been shown to predict therapy type and the likelihood of several adverse perinatal outcomes in women with GDM^a^. |
|  | Ducarme et al. 2019 ^6^ | France | Secondary analysis of a prospective observational study | Development and validation | IADSPG^c^ and national guidelines | 1 | 200 | Logistic regression | HbA1c^o^: AUROC^i^ 0.58 (0.48–0.67, 95% CI^l^),  Sensitivity: 29.7%,  Specificity: 87.7%,  PPV^j^ 59.4%.  1hr OGTT^m^:  AUROC^i^ 0.62 (0.50–0.74, 95% CI^l^),  Sensitivity: 29.7,  Specificity: 66.7%,  PPV^j^ 44.0%. | To determine maternal and biological parameters at diagnosis of GDM^a^ as predictors of antenatal insulin therapy for glycaemic control. | HbA1c^o^ at diagnosis of GDM^a^ and elevated 1-hour OGTT^m^ were independent predictors of insulin for glycaemic control. |
|  | Eleftheriades et al. 2021 ^7^ | Greece | Prospective cohort study | Development and validation | IADSPG^c^ | 8 | 775 | CART^e^ | AUROC^i^ 0.75 (0.7-0.78, 95% CI^l^) | To develop a predictive model for the necessity of insulin treatment in women with GDM^a^. | Overweight women with an abnormal baseline blood glucose at OGTT^m^ have a high likelihood of insulin treatment. |
|  | Ford et al. 2022 ^8^ | Australia | Retrospective cohort study | Development and validation | ADIPS^b^ | 1 | 2048 | Logistic regression | AUROC^i^ 0.744 (0.720-0.767, 95% CI^l^) | To identify predictors of insulin therapy in women diagnosed with GDM^a^ once an OGTT^m^ is performed during pregnancy. | Reasonable prediction of the need for insulin use can be achieved with information routinely collected in antenatal care. |
|  | Harper et al. 2016 ^9^ | United States | Retrospective cohort study | Development and validation | Carpenter and Coustan criteria | 6 | 360 | Logistic regression | AUROC^i^ 0.86,  Sensitivity 39.7%, Specificity 97.5% | To develop a prediction model to identify women with GDM^a^ who require insulin to achieve glycaemic control. | Women with GDM^a^ who will require insulin can be identified at the initiation of pharmacological therapy. |
|  |  |  |  |  |  |  |  | Logistic regression | AUROC^i^ 0.87,  Sensitivity 30.8%,  Specificity 98.8% |  |  |
|  | Khin et al. 2018 ^10^ | United Kingdom | Retrospective cohort study | Development | IADSPG^c^ | 3 | 228 | Logistic regression | Specificity 64%, Sensitivity 87%, PPV^j^ 74%,  NPV^k^ 70%, | To identifying the characteristics of these women with GDM^a^ may help insulin in addition to metformin define optimal therapeutic strategy. | Women with higher fasting glucose levels have a higher chance of necessitating insulin in later pregnancies. |
|  | Nishikawa et al. 2018 ^11^ | Japan | Retrospective cohort study | Development | IADSPG^c^ | 1 | 529 | Logistic regression | AUROC^i^ 0.723,  Sensitivity 67.3%,  Specificity 67.3% | To identify factors predicting the need for insulin therapy in GDM^a^ patients. | Antepartum 1-h glucose levels in a 75-g OGTT^m^ were a predictor of the need for insulin therapy in pregnancy. |
|  | Souza et al. 2019 ^12^ | Brazil | Retrospective cohort study | Development and validation | IADSPG^c^ | 3 | 408 | Logistic regression | Specificity 90%, Sensitivity 36%, PPV^j^ 66%,  NPV^k^ 74%, Accuracy 74% | To evaluate risk factors and propose a model for the prediction of insulin requirement during the treatment of early-diagnosed GDM^a^. | The need for insulin therapy in women with early diagnosis of GDM^a^ can be predicted by a logistic regression model. |
|  | Tamagawa et al. 2021 ^13^ | Japan | Retrospective cohort study | Development | IADSPG^c^ and national guidelines | 9 | 388 | Logistic regression | Pre-pregnancy BMI^n^: AUROC^i^ 0.62,  Sensitivity 63.3%,  Specificity 57.8%,  PPV^j^ 54.4%,  NPV^k^ 73.8%.  Fasting plasma glucose: AUROC^i^ 0.52,  Sensitivity 20.1%,  Specificity 92.0%,  PPV^j^ 58.3%,  NPV^k^ 82.3%.  1hr plasma glucose: AUROC^i^ 0.77,  Sensitivity 71.2%,  Specificity 74.7%,  PPV^j^ 61.1%,  NPV^k^ 84.3%.  2hr plasma glucose:  AUROC^i^ 0.75,  Sensitivity 78.4%,  Specificity 64.7%,  PPV^j^ 61.1%,  NPV^k^ 84.3%. | To elucidate factors that predict patients with GDM^a^ diagnosed before 24 gestational weeks who require insulin therapy later during pregnancy. | Women with an early diagnosis of GDM^a^, a pre-pregnancy BMI^n^ ≥25 kg/m^2^, and a family history of diabetes are more likely to require insulin therapy later during pregnancy. |
|  | Tang et al. 2019 ^14^ | China | Retrospective cohort study | Development | IADSPG^c^ and national guidelines | 3 | 534 | Logistic regression | Fasting plasma glucose 5.7 mmol/L: AUROC^i^ 07.88, (0.704–0.872 CI^l^)  Sensitivity 59.6%,  Specificity 89.9%,  1hr plasma glucose, 11.4 mmol/L: AUROC^i^ 0.642, (0.540-0.744 CI^l^)  Sensitivity 34.0%,  Specificity 94.4%,  HbA1c^o^ 5.3% AUROC^i^ 0.683, (0.587-0.779 CI^l^)  Sensitivity 59.6%,  Specificity 70.8%, | To investigate the potential predictors of insulin treatment during pregnancy and abnormal postpartum glucose metabolism in GDM^a^. | Patients with fasting plasma glucose >5.7 mmol/L, 1 h plasma glucose >11.4 mmol/L, or HbA1c^o^ >5.3% of GDM^a^ diagnoses required insulin treatment. With fasting plasma glucose at GDM^a^ diagnosis was the most important predictor. |
|  | Watanabe et al. 2016 ^15^ | Japan | Retrospective cohort study | Development | IADSPG^c^ | 6 | 37 | Logistic regression | 1hr plasma glucose, 10.25 mmol/L:  AUROC^i^ 0.872,  Sensitivity 100%,  Specificity 77.8%.  2hr plasma glucose, 8.75 mmol/L: AUROC^i^ 0.756,  Sensitivity 70%,  Specificity 70.4%.  75g OGTT^m^ 1.5:  AUROC^i^ 0.783,  Sensitivity 80%,  Specificity 74.1% | To investigate the clinical characteristics of patients with GDM^a^ to identify risk factors for antenatal insulin treatment. | 1- h plasma glucose levels in 75 g OGTT^m^ are useful parameters in predicting the requirement for insulin in GDM^a^. |
|  | Weschenfelder et al. 2021 ^16^ | Germany | Retrospective cohort study | Development | IADSPG^c^ and national guidelines | 5 | 454 | Logistic regression | Fasting plasma glucose 5.5 mmol/L:  AUROC^i^ 0.643 (0.590-0.696, 95% CI^l^)  Specificity 84.4%, Sensitivity 42.5%, PPV^j^ 69.3%,  NPV^k^ 63.9%.  1hr glucose 10.6 mmol/L AUROC^i^ 0.643 (0.582-0.686, 95% CI^l^)  Specificity 76.7%, Sensitivity 45.3%, PPV^j^ 55.9%,  NPV^k^ 68.3%.  HbA1c^o^, 5.4%: AUROC^i^ 0.653 (0.603-0.675, 95% CI^l^)  Specificity 84.4%, Sensitivity 42.5%, PPV^j^ 51.9%,  NPV^k^ 69.4%. | To find predictors of both the general insulin requirement as well as for the described treatment subgroups within women diagnosed with GDM^a^ after 24 weeks of gestation. | Significant cut-offs for insulin dependency were HbA1c^o^ level of 5.4%, fasting plasma glucose of 5.5 mmol/L and 1 hr glucose of 10.6 mmol/L. |
|  |  |  |  |  |  |  |  | Logistic regression | 1hr glucose, 10.6 mmol/L: AUROC^i^ 0.63 (0.55-0.711, 95% CI^l^)  Specificity 70.2%, Sensitivity 51.1%, PPV^j^ 15.9%,  NPV^k^ 92.9%.  HbA1c^o^, 5.4%: AUROC^i^ 0.6 (0.525-0.675, 95% CI^l^)  Specificity 60.1%, Sensitivity 57.8%, PPV^j^ 13.8%,  NPV^k^ 92.8%. |  |  |
|  |  |  |  |  |  |  |  | Logistic regression | Fasting plasma glucose, 5.2 mmol/L:  AUROC^i^ 0.613 (0.543-0.682, 95% CI^l^)  Specificity 48.9%, Sensitivity 70.5%, PPV^j^ 17.6%,  NPV^k^ 91.4%. |  |  |
|  |  |  |  |  |  |  |  | Logistic regression | Fasting plasma glucose, 5.6mmol/L: AUROC^i^ 0.723 (0.653-0.793, CI^l^)  Specificity 83.7%, Sensitivity 56.2%, PPV^j^ 39.8%,  NPV^k^ 90.9%.  1hr glucose, 10.7 mmol/L:  AUROC^i^ 0.655 (0.583-0.727),9 5% CI^l^)  Specificity 73.5%, Sensitivity 49.3%, PPV^j^ 26.3%,  NPV^k^ 88.3%.  HbA1c^o^, 5.4%: AUROC^i^ 0.734 (0.672-0.796, 95% CI^l^)  Specificity 63.8%, Sensitivity 69.9%, PPV^j^ 27%,  NPV^k^ 91.7%.  Abdominal circumference 69^th^ percentile: AUROC^i^ 0.662 (0.591-0.733, 95% CI^l^)  Specificity 60.3%, Sensitivity 69%, PPV^j^ 26%,  NPV^k^  89.8%. |  |  |
|  | Zaccara et al. 2023 ^17^ | Brazil | Retrospective cohort study | Development | ADA^d^ | 8 | 869 | Logistic regression | AUROC^i^ 0.77 (0.72-0.81, 95% CI^l^) | To identify risk factors associated with insulin need in women with GDM^a^. | Regularly collected data from patients can calculate the risk of a woman with GDM^a^ diagnosed in OGTT^m^ needing insulin. |

^a^GDM Gestational diabetes mellitus

^b^ADIPS Australasian Diabetes in Pregnancy Society

^c^IADPSG International Association of the Diabetes and Pregnancy Study Group

^d^ADA American Diabetes Association

^e^CART Classification and regression Tree

^f^LASSO Least absolute shrinkage and selection operator

^g^Simple super learner could have been included response-mean, least absolute shrinkage and selection operator regression, and classification and regression tree

^h^Complex super learner could have been response-mean, least absolute shrinkage and selection operator regression, classification and regression tree, random forest, and extreme gradient boosting

^i^AUROC Area under the receiver operating characteristics

^j^PPV Positive predictive value

^k^NPV Negative predictive value

^l^CI Confidence interval

^m^OGTT Oral glucose tolerance test

^n^BMI Body mass index

^o^HbA1c glycohemoglobin, hemoglobin

1. Feghali MN, Abebe KZ, Comer DM, Caritis S, Catov JM, Scifres CM. Response to Medical Nutritional Therapy and Need for Pharmacological Therapy in Women with Gestational Diabetes. *Am J Perinatol*. Oct 2019;36(12):1250-1255. doi:10.1055/s-0038-1676615

2. Liao LD, Ferrara A, Greenberg MB, et al. Development and validation of prediction models for gestational diabetes treatment modality using supervised machine learning: a population-based cohort study. *BMC Medicine*. 2022;20(307)doi:10.1186/s12916-022-02499-7

3. Velardo C, Clifton D, Hamblin S, Khan R, Tarassenko L, Mackillop L. Toward a Multivariate Prediction Model of Pharmacological Treatment for Women With Gestational Diabetes Mellitus: Algorithm Development and Validation. *Journal of Medical Internet Research*. 2021;23(3):e21435. doi:10.2196/21435

4. Yerlikaya G, Falcone V, Stopp T, et al. To Predict the Requirement of Pharmacotherapy by OGTT Glucose Levels in Women with GDM Classified by the IADPSG Criteria. *Journal of Diabetes Research*. 2018;2018:1-6. doi:10.1155/2018/3243754

5. Barnes RA, Wong T, Ross GP, et al. A novel validated model for the prediction of insulin therapy initiation and adverse perinatal outcomes in women with gestational diabetes mellitus. *Diabetologia*. 2016;59(11):2331-2338. doi:10.1007/s00125-016-4047-8

6. Ducarme G, Desroys Du Roure F, Grange J, Vital M, Le Thuaut A, Crespin‐Delcourt I. Predictive factors of subsequent insulin requirement for glycemic control during pregnancy at diagnosis of gestational diabetes mellitus. *International Journal of Gynecology and Obstetrics*. 2019;144(3):265-270. doi:10.1002/ijgo.12753

7. Eleftheriades M, Chatzakis C, Papachatzopoulou E, et al. Prediction of insulin treatment in women with gestational diabetes mellitus. *Nutrition and Diabetes*. 2021;11(1)doi:10.1038/s41387-021-00173-0

8. Ford HL, Champion I, Wan A, Reddy M, Mol BW, Rolnik DL. Predictors for insulin use in gestational diabetes mellitus. *European Journal of Obstetrics & Gynecology and Reproductive Biology*. 2022/05/01/ 2022;272:177-181. doi:<https://doi.org/10.1016/j.ejogrb.2022.03.025>

9. Harper LM, Glover AV, Biggio JR, Tita A. Predicting failure of glyburide therapy in gestational diabetes. *Journal of Perinatology*. 2016;36(5):347-351. doi:10.1038/jp.2015.216

10. Khin MO, Gates S, Saravanan P. Predictors of metformin failure in gestational diabetes mellitus (GDM). *Diabetes & Metabolic Syndrome: Clinical Research & Reviews*. 2018/05/01/ 2018;12(3):405-410. doi:<https://doi.org/10.1016/j.dsx.2018.01.003>

11. Nishikawa T, Ono K, Hashimoto S, et al. One-hour oral glucose tolerance test plasma glucose at gestational diabetes diagnosis is a common predictor of the need for insulin therapy in pregnancy and postpartum impaired glucose tolerance. *Journal of Diabetes Investigation*. 2018;9(6):1370-1377. doi:10.1111/jdi.12848

12. Souza ACRLA, Costa RA, Paganoti CF, et al. Can we stratify the risk for insulin need in women diagnosed early with gestational diabetes by fasting blood glucose? *The Journal of Maternal-Fetal & Neonatal Medicine*. 2019;32(12):2036-2041. doi:10.1080/14767058.2018.1424820

13. Tamagawa M, Kasuga Y, Saisho Y, et al. Predictors of later insulin therapy for gestational diabetes diagnosed in early pregnancy. *Endocrine Journal*. 2021;68(11):1321-1328. doi:10.1507/endocrj.ej21-0118

14. Tang L, Xu S, Li P, Li L. Predictors of Insulin Treatment During Pregnancy and Abnormal Postpartum Glucose Metabolism in Patients with Gestational Diabetes Mellitus. *Diabetes, Metabolic Syndrome and Obesity: Targets and Therapy*. 2019;12:2655-2665. doi:10.2147/dmso.s233554

15. Watanabe M, Katayama A, Kagawa H, Ogawa D, Wada J. Risk Factors for the Requirement of Antenatal Insulin Treatment in Gestational Diabetes Mellitus. *Journal of Diabetes Research*. 2016;2016:1-6. doi:10.1155/2016/9648798

16. Weschenfelder F, Lohse K, Lehmann T, Schleußner E, Groten T. Predictors of Treatment Requirements in Women with Gestational Diabetes: A Retrospective Analysis. *Journal of Clinical Medicine*. 2021;10(19):4421. doi:10.3390/jcm10194421

17. Zaccara TA, Mikami FCF, Paganoti CF, Francisco RPV, Costa RA. Predicting insulin use among women with gestational diabetes diagnosed in oral glucose tolerance test. *BMC Pregnancy and Childbirth*. 2023;23(1)doi:10.1186/s12884-023-05746-8
