# Supplementary material for: The use of machine learning to predict pharmacological therapy in gestational diabetes: A scoping review
Source: Diabet Med. 2025 Nov 18;43(2):e70171. doi: 10.1111/dme.70171 (PMC12857867; doi:10.1111/dme.70171)
Supplement: Supplementary file 3 — Data S3. [file DME-43-e70171-s005.docx]

Supplementary material 3

Performance metrics

**Whole model algorithm performance, median (range)**

|  | AUROC^a^ | Sensitivity, % | Specificity, % | PPV^b^ | NPV^c^ |
| --- | --- | --- | --- | --- | --- |
| Any algorithm | 0.75 (0.61-0.93) | 59.6 (9.3-90.0) | 81.2 (36.0-91.5) | 67.5 (17.6-92.5) | 70.0 (58.0-74.0) |
| Logistic regression | 0.75 (0.63-0.87) | 59.6 (9.3-90.0) | 89.9 (36-99.4) | 67.54 (58.0-74.0) | 70.0 (58.0-74.0) |
| CART^d^ | 0.74 (0.61-0.79) | - | - | - | - |
| LASSO^e^ | 0.74 (0.67-0.84) | - | - | - | - |
| Simple super learner^f^ | 0.74 (0.67-0.85) | - | - | - | - |
| Complex super learner^g^ | 0.82 (0.64-0.93) | - | - | - | - |

^a^AUROC Area under the receiver operating curve

^b^PPV Positive predictive value

^c^NPV Negative predictive value

^d^CART Classification and regression trees

^e^LASSO Least absolute shrinkage and selection operator

^f^ Simple super learner could have been included response-mean, least absolute shrinkage and selection operator regression, and Classification and regression tree

^g^Complex super learner could have been response-mean, least absolute shrinkage and selection operator regression, Classification and regression tree, random forest, and extreme gradient boosting

**Whole model algorithm performance predicting insulin, median (range)**

|  | AUROC^a^ | Sensitivity, % | Specificity, % | PPV^b^ | NPV^c^ |
| --- | --- | --- | --- | --- | --- |
| Any algorithm | 0.74 (0.71 – 0.87) | 54.3 (9.3 -90/0) | 90.65 (36.0 – 99.4) | 70/0  917.6-87.6) | 70.0 (69.9-74.0) |
| Logistic regression | 0.76 (0.71-0.87) | 54.3 (9.3-90.0) | 90.7 (36.0-99.4) | 70.0 (17.6 -87.6) | 70 (69.9-74) |
| CART^d^ | 0.74* | - | - | - | - |

^a^AUROC Area under the receiver operating curve

^b^PPV Positive predictive value

^c^NPV Negative predictive value

^d^CART Classification and regression trees

* only appeared in one model

**Whole model algorithm performance predicting pharmacological therapy, median (range)**

|  | AUROC^a^ | Sensitivity, % | Specificity, % | PPV^b^ | NPV^c^ |
| --- | --- | --- | --- | --- | --- |
| Any algorithm | 0.75 (0.61 – 0.93) | 72.2* | 86.8* | 92.5* | 58* |
| Logistic regression | 0.75 (0.63-0.83) | 72.2* | 86.8* | 92.5* | 58.0* |
| CART^d^ | 0.68 (0.61-0.79) | - | - | - | - |
| LASSO^e^ | 0.74 (0.67-0.85) | - | - | - | - |
| Simple super learner^f^ | 0.74 (0.67-0.85) | - | - | - | - |
| Complex super learner^g^ | 0.82 (0.64-0.94) | - | - | - |  |

^a^AUROC Area under the receiver operating curve

^b^PPV Positive predictive value

^c^NPV Negative predictive value

^d^CART Classification and regression trees

^e^LASSO Least absolute shrinkage and selection operator

^f^ Simple super learner could have been included response-mean, least absolute shrinkage and selection operator regression, and Classification and regression tree

^g^Complex super learner could have been response-mean, least absolute shrinkage and selection operator regression, Classification and regression tree, random forest, and extreme gradient boosting

* only appeared in one model

**Individual model algorithm performance, median (range) [All predicting insulin and used logistic regression]**

|  | AUROC^a^ | Sensitivity, % | Specificity, % | PPV^b^ | NPV^c^ |
| --- | --- | --- | --- | --- | --- |
| HbA1c | 0.63 (0.58-0.73) | 62.4 (29.7 -  69.9) | 62.0 (54.7-  87.7) | 39.5 (13.8 -59.4) | 69.4 (61.7-  92.8) |
| Fasting 75g OGTT | 0.64 (0.52-0.72) | 42.5 (20.1-  56.2) | 84.4 (83.7-  92.0) | 58.3 (39.8-  63.9) | 82.3 (69.3 -  90.9) |
| 1hr 75G OGTT | 0.65 (0.62-0.88) | 50.2 (29.7-  100) | 74.1 (66.7-  77.8) | 44.0 (15.9 -  61.1) | 86.3 (68.3-  92.9) |
| 2hr 75g OGTT | 0.75 (0.75 -  0.76) | 74.2 (70. -  78.4) | 67.6 (64.7-  70.4) | 55.3* | 84.3 (84.3-  84.3) |
| Number of abnormal values in 75g OGTT * | 0.78 | 80.0 | 74.1 | - | - |
| Pre-pregnancy BMI* | 0.62 | 63.3 | 57.8 | 55.4 | 73.8 |
| Abdomincal Circumference percentile* | 0.66 | 60.3 | 67.2 | 26.0 | 89.3 |

^a^AUROC Area under the receiver operating curve

^b^PPV Positive predictive value

^c^NPV Negative predictive value

* only appeared in one model

**Whole model performance within class imbalance, median (range)**

| **Predictive group, %** | **AUROC** |
| --- | --- |
| 10-20 | 0.76 (0.72-0.87) |
| 21-30 | 0.78 (0.71-0.86) |
| 31-40 | 0.76 (0.61-0.93) |
| 41-50 | 0.72 (0.71-0.78) |
| >51 | 0.77 (0.70-0.83) |

^a^AUROC Area under the receiver operating curve

**Whole model performance within GDM diagnostic criteria, median (range)**

|  | AUROC^a^ | Sensitivity, % | Specificity, % | PPV^b^ | NPV^c^ |
| --- | --- | --- | --- | --- | --- |
| ADA^d*^ | 0.77 | - | - | - | - |
| ADIPS^e^ | 0.72 (0.71 – 0.74) | - | - | - | - |
| Carpenter and Coustan criteria | 0.77 (0.61 – 0.93) | 36.7 (30.8 – 98.8) | 97.5 (86.6 – 92.5) | 92.5* | 58* |
| IADSPG^f^ | 0.74 (0.71 – 0.78) | 67.3 (64.0 -90.0) | 67.3 (36.0 - 87.0) | 70.0 (66.0 – 74.0) | 72.0 (70.0 -74.0) |
| IADSPG^f^ and national guidelines* | 0.80 | - | - | - | - |

^a^AUROC Area under the receiver operating curve

^b^PPV Positive predictive value

^c^NPV

^d^ADA American Diabetes Association

^e^ADIPS Australasian Diabetes in Pregnancy Society

^f^IADPSG International Association of the Diabetes and Pregnancy Study Group

* only appeared in one model
