# Supplementary material for: The use of machine learning to predict pharmacological therapy in gestational diabetes: A scoping review
Source: Diabet Med. 2025 Nov 18;43(2):e70171. doi: 10.1111/dme.70171 (PMC12857867; doi:10.1111/dme.70171)
Supplement: Supplementary file 4 — Data S4. [file DME-43-e70171-s003.docx]

Supplementary material 4

Risk of bias and applicability assessment

Table 3 PROBAST risk of bias and applicability assessment

|  | Author | Model | Risk of bias | | | | Applicability | | | Overall | |
| --- | --- | --- | --- | --- | --- | --- | --- | --- | --- | --- | --- |
|  |  | Model description | Participants | Predictors | Outcome | Analysis | Participants | Predictors | Outcome | Risk of bias | Applicability |
| Predicting pharmacological therapy | Feghali et al. 2019 ^34^ | Without SMBG^a^ | **-** | **+** | **+** | **-** | **+** | **+** | **+** | **-** | **?** |
|  |  | With SMBG^a^ | **-** | **+** | **+** | **-** | **+** | **+** | **+** | **-** | **?** |
|  | Liao et al. 2022 ^22^ | CART^b^ L1^c^ | **-** | **+** | **+** | **+** | **-** | **+** | **?** | **-** | **-** |
|  |  | CART^b^ L1-2^d^ | **-** | **+** | **+** | **+** | **-** | **+** | **?** | **-** | **-** |
|  |  | CART^b^ L1-3^e^ | **-** | **+** | **+** | **+** | **-** | **+** | **+** | **-** | **-** |
|  |  | CART^b^ L1-4^f^ | **-** | **+** | **+** | **+** | **-** | **+** | **+** | **-** | **-** |
|  |  | LASSO^g^ L1^c^ | **-** | **+** | **+** | **+** | **-** | **+** | **?** | **-** | **-** |
|  |  | LASSO^g^ L1-2^d^ | **-** | **+** | **+** | **+** | **-** | **+** | **?** | **-** | **-** |
|  |  | LASSO^g^ L1-3^e^ | **-** | **+** | **+** | **+** | **-** | **+** | **+** | **-** | **-** |
|  |  | LASSO^g^ L1-4^f^ | **-** | **+** | **+** | **+** | **-** | **+** | **+** | **-** | **-** |
|  |  | SL^h^ L1^c^ | **-** | **+** | **+** | **+** | **-** | **+** | **?** | **-** | **-** |
|  |  | SL^h^ L1-2^d^ | **-** | **+** | **+** | **+** | **-** | **+** | **?** | **-** | **-** |
|  |  | SL^h^ L1-3^e^ | **-** | **+** | **+** | **+** | **-** | **+** | **?** | **-** | **-** |
|  |  | SL^h^ L1-4^f^ | **-** | **+** | **+** | **+** | **-** | **+** | **+** | **-** | **-** |
|  |  | CL^i^ L1^c^ | **-** | **+** | **+** | **+** | **-** | **+** | **?** | **-** | **-** |
|  |  | CL^i^ L1-2^d^ | **-** | **+** | **+** | **+** | **-** | **+** | **?** | **-** | **-** |
|  |  | CL^i^ L1-3^e^ | **-** | **+** | **+** | **+** | **-** | **+** | **?** | **-** | **-** |
|  |  | CL^i^ L1-4^f^ | **-** | **+** | **+** | **+** | **-** | **+** | **+** | **-** | **-** |
|  |  | LR^j^ L1^c^ | **-** | **+** | **+** | **+** | **-** | **+** | **?** | **-** | **-** |
|  |  | LR^j^ L1-2^d^ | **-** | **+** | **+** | **+** | **-** | **+** | **?** | **-** | **-** |
|  |  | LR^j^ L1-3^e^ | **-** | **+** | **+** | **+** | **-** | **+** | **+** | **-** | **-** |
|  |  | LR^j^ L1-4^f^ | **-** | **+** | **+** | **+** | **-** | **+** | **+** | **-** | **-** |
|  | Velardo et al. 2021 ^28^ |  | **+** | **-** | **+** | **+** | **-** | **-** | **+** | **-** | **-** |
|  | Yerlikaya et al. 2018 ^23^ | Using OGTT^k^ | **+** | **+** | **+** | **-** | **+** | **+** | **+** | **?** | **+** |
|  |  | Using clinical variables | **+** | **+** | **+** | **-** | **+** | **+** | **+** | **?** | **+** |
|  |  | Using OGTT^k^ and clinical variables | **+** | **+** | **+** | **-** | **+** | **+** | **+** | **?** | **+** |
|  |  | Random forest | **+** | **+** | **+** | **-** | **+** | **+** | **+** | **-** | **?** |
| Predicting insulin | Barnes et al. 2016 ^35^ |  | **+** | **+** | **+** | **?** | **+** | **+** | **?** | **-** | **?** |
|  | Ducarme et al. 2019 ^24^ |  | **-** | **+** | **+** | **?** | **-** | **+** | **+** | **-** | **-** |
|  | Eleftheriades et al. 2021 ^26^ |  | **-** | **+** | **+** | **-** | **+** | **-** | **+** | **-** | **?** |
|  | Ford et al. 2022 ^36^ |  | **+** | **+** | **+** | **+** | **+** | **+** | **+** | **+** | **?** |
|  | Harper et al. 2016 ^33^ | Including pre-diabetes | **-** | **+** | **+** | **-** | **+** | **+** | **-** | **-** | **-** |
|  |  | Excluding pre-diabetes | **-** | **+** | **+** | **?** | **+** | **+** | **-** | **-** | **-** |
|  | Khin et al. 2018 ^27^ |  | **?** | **+** | **+** | **-** | **+** | **+** | **+** | **-** | **-** |
|  | Nishikawa et al. 2018 ^31^ |  | **+** | **+** | **+** | **?** | **+** | **+** | **+** | **-** | **-** |
|  | Souza et al. 2019 ^37^ |  | **+** | **+** | **+** | **?** | **+** | **+** | **+** | **?** | **+** |
|  | Tamagawa et al. 2021 ^32^ |  | **-** | **+** | **+** | **-** | **+** | **+** | **+** | **-** | **+** |
|  | Tang et al. 2019 ^29^ |  | **?** | **+** | **+** | **-** | **+** | **+** | **+** | **-** | **+** |
|  | Watanabe et al. 2016 ^30^ |  | **?** | **+** | **+** | **-** | **+** | **+** | **+** | **-** | **?** |
|  | Weschenfelder et al. 2021 ^25^ | Predicting insulin | **+** | **+** | **+** | **-** | **+** | **+** | **+** | **-** | **?** |
|  |  | Predicting Bolus insulin | **+** | **+** | **+** | **-** | **+** | **+** | **+** | **-** | **?** |
|  |  | Predicting basal insulin | **+** | **+** | **+** | **-** | **+** | **+** | **+** | **-** | **?** |
|  |  | Predicting multiple injections | **+** | **+** | **+** | **-** | **+** | **+** | **+** | **-** | **?** |
|  | Zaccara et al. 2023 ^38^ |  | **+** | **+** | **+** | **-** | **+** | **+** | **+** | **-** | **?** |

**+**: Low risk of bias/concern about applicability, **-**: high risk of bias/concern about applicability, **?**: unclear risk of bias/concern about applicability

^a^SMBG Self monitoring blood glucose

^b^CART Classification and regression Tree

^c^L1 Data from 1-year preconception to last menstrual period

^d^L1-2 Data from 1-year preconception to last menstrual period until last menstrual period to before diagnosis of GDM

^e^L1-3 Data from 1-year preconception until the time of diagnosis of GDM

^f^L1-4 L1 Data from 1-year preconception to last menstrual period until 1 week after diagnosis of GDM (including self-monitoring blood glucose data)

^g^LASSO Least absolute shrinkage and selection operator

^h^SL Simple super learner could have been included response-mean, least absolute shrinkage and selection operator regression, and classification and regression tree

^i^CL Complex super learner could have been response-mean, least absolute shrinkage and selection operator regression, classification and regression tree, random forest, and extreme gradient boosting

^j^LR Logistic regression

^k^OGTT Oral glucose tolerance test
