# Supplementary material for: The use of machine learning to predict pharmacological therapy in gestational diabetes: A scoping review
Source: Diabet Med. 2025 Nov 18;43(2):e70171. doi: 10.1111/dme.70171 (PMC12857867; doi:10.1111/dme.70171)
Supplement: Supplementary file 5 — Data S5. [file DME-43-e70171-s004.docx]

Supplementary material 5

Sensitivity analysis

Liao et al. (2022)^1^, presented 20 studies, (45.5%) of the included models, therefore a sensitivity analysis was conducted.

| **Topic** | **Before (including Liao et al. (2022)** | **After sensitivity analysis (excluding Liao et al. (2022))** |
| --- | --- | --- |
| Number of included models | 44 | 24 |
| Prediction groups | Predicting insulin: 38.6%, 17/44  Predicting pharmacological therapy: 61.4%, 27/44 | Predicting insulin: 70.8%, 17/24  Predicting pharmacological therapy: 29.2%, 7/24 |
| Study period (median (range)) | 5 years (range 1-23 years) | 5 years (range 1-23 years) |
| Number of participants (median (range)) | 1919 participants (range 37 - 30,474) | 304 participants (range 37-2217) |
| Percentage of participants in control or predictive group  (median (range)) | Control group: 61.2% (range 30.2-89.2%)  Predictive group: 38.8% (range 10.8-69.8%) | Control group: 65.1% (range 30.2-89.2)  Predictive group: 35.0% (range 10.8-69.8%) |
| Algorithms used (percentage, frequency) | Logistic regression (59.1%, 26/44),  CART^a^ (11.4%, 5/44),  LASSO^b^ (9.1%, 4/44),  Simple super learner (either response-mean, LASSO^b^, and CART^a^) (9.1%, 4/44),  Complex super learner (either response-mean, LASSO^b^, CART^a^, random forest, or extreme gradient boosting), (9.1%, 4/44) | Logistic regression (97.7%, 22/24),  CART^a^ (4.2%, 1/24),  Random forest (4.2%, 1/24) |
| Frequently used variables overall (percentage, frequency) | History of GDM^c^ (47.7%, 21/44), Gestational week at GDM^c^ diagnosis (45.5%, 20/44), Pregestational BMI^d^ (40.9%, 18/44), and  Maternal age (38.6%, 17/44). | 1hr 75g OGTT^e^ (58.3% 14/24),  Fasting 75g OGTT^e^ (54.2%, 13/24), Maternal age (41.7%, 10/24)  Gestational week at GDM^c^ diagnosis (41.7%, 10/24) |
| Frequently used variables predicting pharmacological therapy (percentage, frequency) | History of GDM^c^ (51.9%, 14/27), Gestational week at GDM^c^ Diagnosis (51.9%, 14/27), Pregestational BMI^d^ (48.1%, 13/27), and  Maternal age (41.8%, 13/27) | Maternal age (85.7%, 6/7), Gestational week at GDM^c^ diagnosis (57.1%, 4/7),  Pregestational BMI^d^ (57.1%, 4/7) and  Parity (57.1%, 4/7) |
| Performance metrics used (percentage, frequency) | AUROC^f^ (95.5%, 42/44),  Sensitivity and specificity (36.4%, 16/44),  PPV^g^ and NPV^h^ (25.0%, 11/44) | AUROC^f^ (91.7%, 22/24),  Sensitivity and specificity: (66.7%, 16/24),  PPV^g^ and NPV^h^ (45.8% ,11/24) |
| Overall AUROC^f^ performance (median (range)) | 0.75 (range 0.61-0.93) | 0.74 (range 0.70-0.87) |
| Logistic regression AUROC^f^ performance (median (range)) | 0.76 (range 0.63-0.87) | 0.76 (range 0.70-0.87) |
| Percentage of models validated, (frequency) | 65.9% (29/44) | 37.5% (9/24) |
| Percentage of overall risk of bias using PROBAST^g^ ^2^, (frequency) | High: 88.6%, (39/44),  Unclear: 9.1%, (4/44),  Low: 2.3%, (1/44) | High: 79.2%, (19/24)  Unclear: 16.7%, (4/24),  Low: 4.2%, (1/24) |
| Percentage of overall concern about applicability using PROBAST^g^ ^2^, (frequency) | High: 59.1%, (26/44),  Unclear: 27.3% (12/44),  Low: 13.6% (6/44) | High: 25.0%, (6/24),  Unclear: 50.0%, (12/24),  Low: 25.0%, (6/24) |

^a^CART Classification and regression trees

^b^LASSO Least absolute shrinkage and selection operator

^c^GDM Gestational diabetes

^d^BMI Body mass index

^e^OGTT Oral glucose tolerance test

^f^AUROC Area under the receiver operating curve

^g^PPV Positive predictive value

^h^NPV Negative predictive value

^i^PROBAST Prediction model Risk Of Bias ASsessment Tool


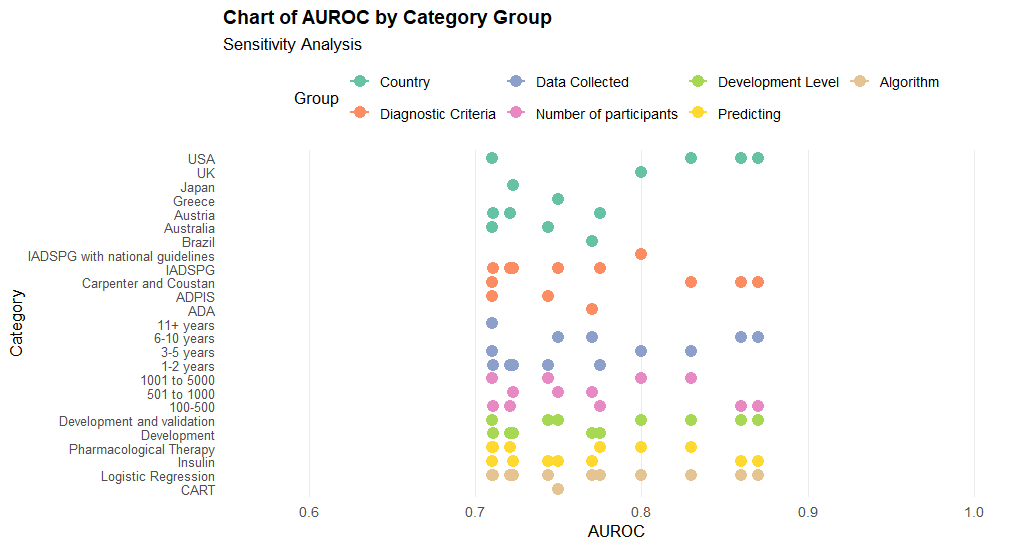


Figure Sensitivity analysis results of AUROC for the whole model performance with the removal of Liao et al. (2022) removed, for categories grouped by country, GDM diagnosis criteria, length of data collection and number of participants within the study, development level of the model, the prediction of the model and the algorithm used.

*AUROC Area Under the Receiver Operating Characteristics, USA – United States of America, UK- United Kingdon, IADSPG – International Association of the Diabetes and Pregnancy Study Group, ADIPS - Australasian Diabetes in Pregnancy Society, ADA - American Diabetes Association, CART Classification and regression Tree, LASSO Least absolute shrinkage and selection operator, Simple super learner could have been included response-mean, least absolute shrinkage and selection operator regression, and classification and regression tree, Complex super learner could have been response-mean, least absolute shrinkage and selection operator regression, Classification and regression tree, random forest, and extreme gradient boosting.*

# Reference

1. Liao LD, Ferrara A, Greenberg MB, et al. Development and validation of prediction models for gestational diabetes treatment modality using supervised machine learning: a population-based cohort study. *BMC Medicine*. 2022;20(307)doi:10.1186/s12916-022-02499-7

2. Wolff RF, Moons KGM, Riley RD, et al. PROBAST: A Tool to Assess the Risk of Bias and Applicability of Prediction Model Studies. *Annals of Internal Medicine*. 2019;170(1):51. doi:10.7326/m18-1376
